# Successful Treatment of Legionnaires’ Disease with Tigecycline in an Immunocompromised Man with a Legion of Antibiotic Allergies

**DOI:** 10.7759/cureus.4577

**Published:** 2019-04-30

**Authors:** Michael Arget, Justin Kosar, Brandon Suen, Shaqil Peermohamed

**Affiliations:** 1 Internal Medicine, University of Saskatchewan College of Medicine, Saskatoon, CAN; 2 Miscellaneous, Saskatchewan Health Authority, Saskatoon, CAN; 3 Internal Medicine / Infectious Disease, University of Saskatchewan College of Medicine, Saskatoon, CAN

**Keywords:** legionella, legionnaires’ disease, pneumonia, tigecycline

## Abstract

Legionella species are Gram-negative bacilli that are relatively rare causes of community-acquired pneumonia but can be associated with significant morbidity and mortality if unrecognized or improperly treated. Limited data exist regarding the use of tigecycline, a third generation glycylcycline, in the treatment of Legionnaires' disease. We present an immunocompromised patient with Legionnaires' disease and allergies to both fluoroquinolones and macrolides, which are first-line treatment options for Legionnaires' disease. He was successfully treated using tigecycline, a third generation glycylcycline, indicating that tigecycline may serve as a safe and effective alternative therapeuticl option for treatment of Legionnaires’ disease.

## Introduction

Legionnaires’ disease is a relatively rare cause of community-acquired pneumonia caused by *Legionella* species; however, 20%-25% of patients who are hospitalized with Legionnaires’ disease require invasive mechanical ventilation and average mortality rates for sporadic disease range from 10% to 15% [1]. Legionnaires’ disease is caused by inhalation of *Legionella* species, which are intracellular, Gram-negative bacilli ubiquitously found in the environment [1-2]. Host risk factors for Legionnaires’ disease include male gender, age older than 50 years, cigarette smoking, diabetes, end-stage renal failure, organ transplantation and immunosuppression, such as glucocorticoids or anti-rejection drugs following organ transplantation [1]. Travel is an important and underappreciated risk factor associated with legionellosis in a community setting [1]. Treatment options for Legionnaires' disease include macrolides, fluoroquinolones, or tetracycline; however, preferred therapies for immunocompromised patients with Legionnaires’ disease include levofloxacin and azithromycin [1-3]. We describe an immunocompromised and severely ill patient with Legionnaires' disease and who also has allergies to both fluoroquinolones and macrolides; he was successfully treated using tigecycline, a third generation glycylcycline, indicating that tigecycline may serve as a safe and effective alternative therapeutic option for treatment of Legionnaires’ disease in select cases.

## Case presentation

A 61-year-old Caucasian man presented to the emergency department in autumn with one week of dyspnea, productive cough, myalgia, and fever. He denied any chest pain or hemoptysis. His past medical history was significant for hypertension, diabetes mellitus, chronic kidney disease, and non-Hodgkin’s lymphoma with receipt of an allogeneic stem cell transplant 13 years prior. Given prior complications due to graft versus host disease, he was receiving prednisone at a maintenance dose of 15 mg daily for several years. He had multiple documented allergies to penicillin, sulfa drugs, macrolides and fluoroquinolones, with reported reactions including rash, hives, and anaphylaxis. Approximately one week prior to the onset of symptoms, he was traveling in the Midwest United States with his partner and staying in various hotels.

Upon arrival to the hospital, he was noted to have a heart rate of 130 beats per minute, a blood pressure of 128/76 mmHg, a respiratory rate of 30 breaths per minute with an oxygen saturation of 89% requiring eight liters of supplementary oxygen, and an oral temperature of 39.8°C (103.6°F). He was in acute respiratory distress and had evident decreased breath sounds and crackles bilaterally. He was noted to have normal heart sounds without any murmurs, rubs, or gallops. He did not have any rash on examination.

**Figure 1:**
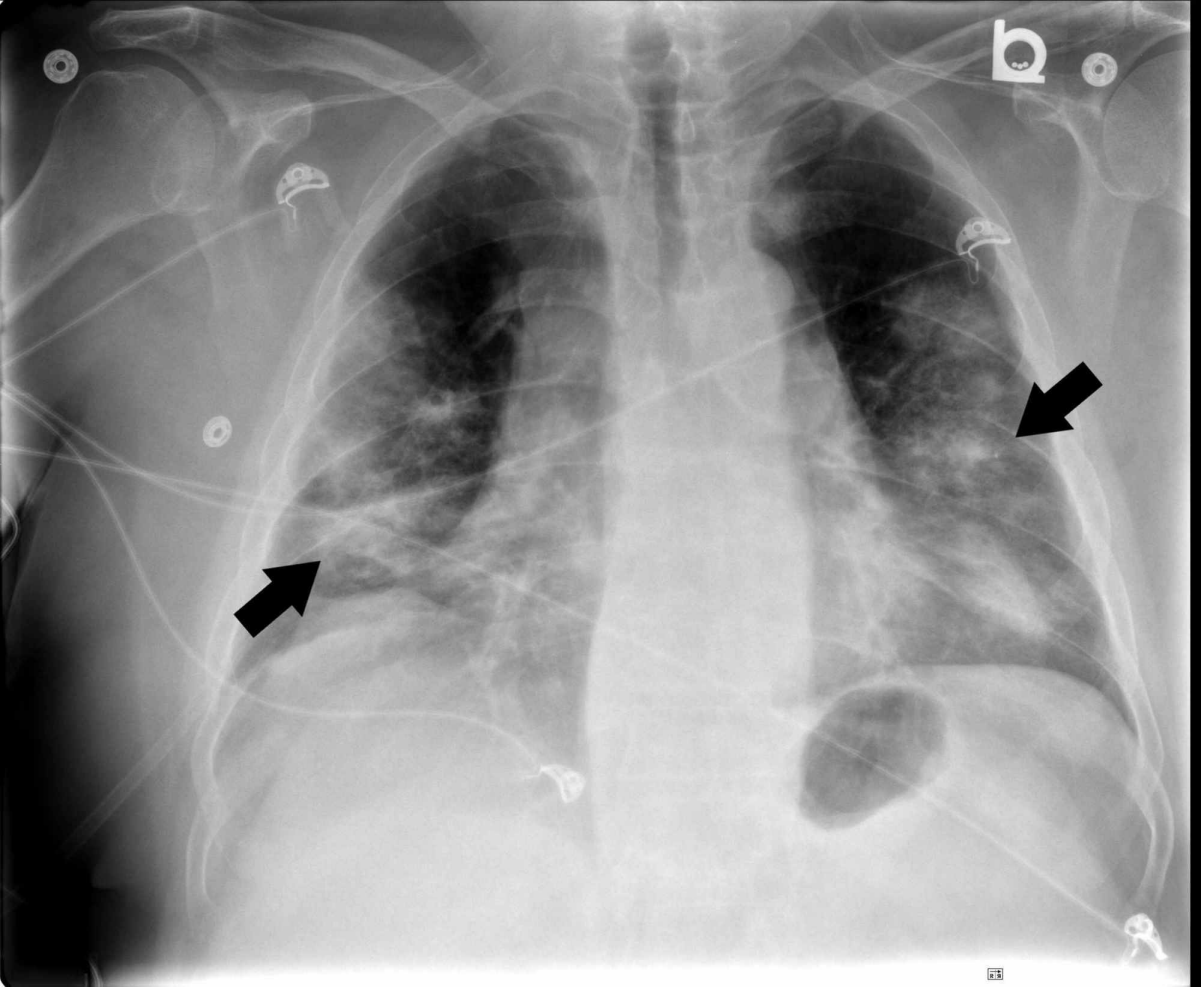
Chest radiograph on admission showing diffuse, bilateral air space opacities (arrows) in the mid and lower lung zones.

Laboratory investigations revealed a normal peripheral leukocyte count of 10.4 x 10^9^ cells/L (10.4 x 10^3^ cells/µL) , decreased hemoglobin of 110 g/L (11.0 g/dL), decreased platelet count of 96 x 109 cells/L (96 x 10^3^ cells/µL), and increased creatinine of 676 µmol/L (7.65 mg/dL). His liver enzymes were normal. His initial chest radiograph revealed diffuse, bilateral air space opacities in the mid and lower lung zones (Figure 1).

Two sets of blood cultures were collected and sputum samples were sent for culture as well as stains and polymerase chain reaction (PCR) testing for *Pneumocystis jirovecii* (*P. jirovecii*). A nasopharyngeal sample was collected for respiratory virus PCR testing for influenza A and B, respiratory syncytial virus A and B, coronavirus, parainfluenza virus, rhinovirus, enterovirus, adenovirus, bocavirus, and metapneumovirus. In addition, a serum cytomegalovirus (CMV) PCR and *Legionella* urine antigen were sent. He was empirically started on meropenem, vancomycin, oseltamivir, and intravenous pentamidine.

**Figure 2:**
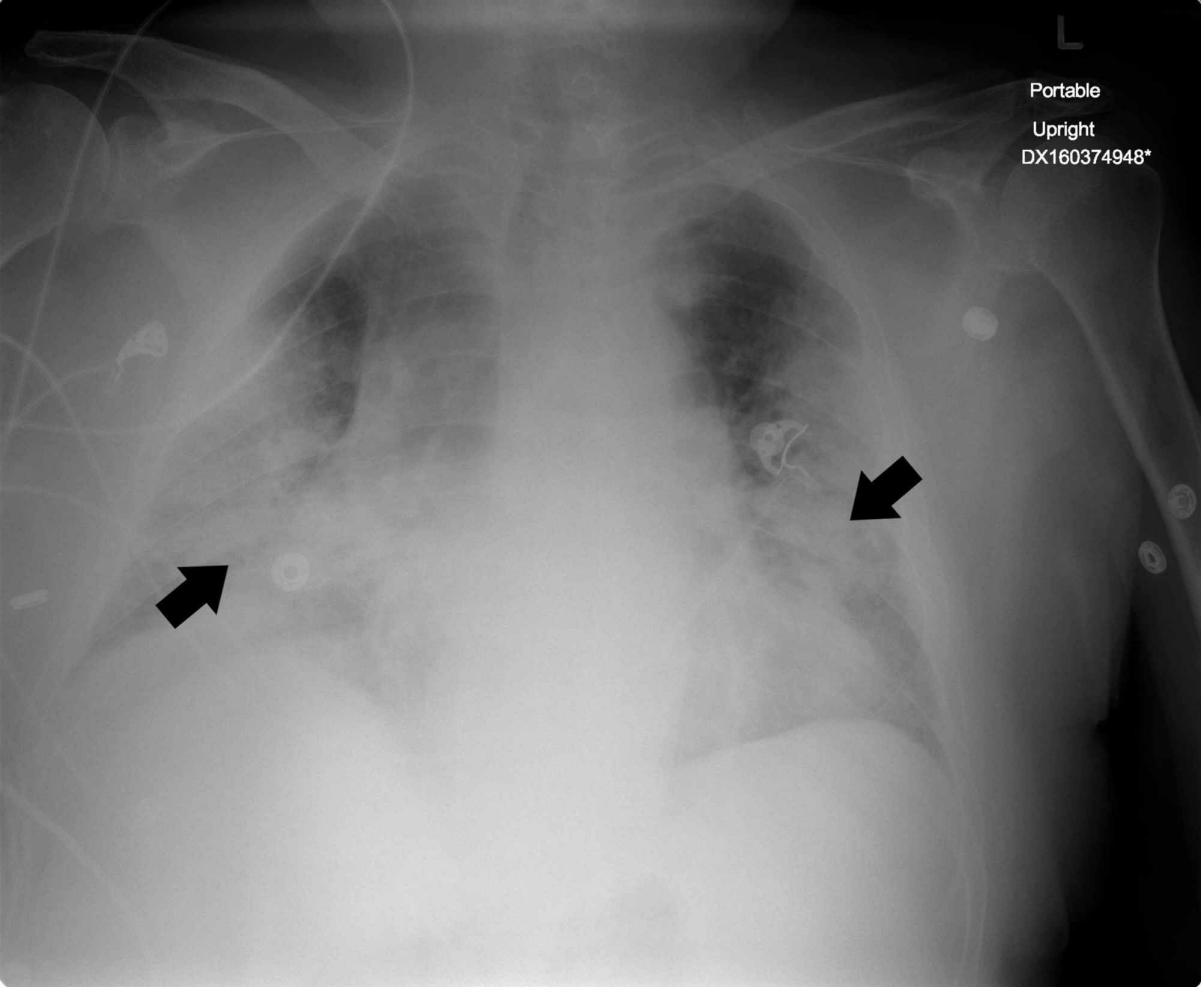
Chest radiograph 48 h following hospital admission, showing worsening bilateral pulmonary opacification (arrows).

Despite initiation of broad spectrum antimicrobials, he continued to deteriorate with increasing oxygen demands, persistent fever, hemodynamic instability, and worsening radiographic infiltrates (Figure 2).

Sputum and blood cultures were negative for any bacterial growth; sputum stains and PCR were negative for *P. jirovecii*. His serum CMV PCR was negative. His nasopharyngeal swab for respiratory virus testing was negative for influenza A and B, respiratory syncytial virus A and B, coronavirus, parainfluenza virus, rhinovirus, enterovirus, adenovirus, bocavirus, and metapneumovirus; however, his *Legionella *urine antigen was positive.

According to his pharmacy records, he had previously received and tolerated a five-day course of moxifloxacin approximately one year prior to this hospital admission. Given his diagnosis of Legionnaires' disease, intravenous moxifloxacin therapy was initiated. However, shortly following receipt of his first dose of moxifloxacin, he developed an allergic reaction with a generalized, erythematous, maculopapular rash and angioedema, necessitating administration of epinephrine.

Given his documented allergies to both fluoroquinolones and macrolides, hemodynamic instability, and concern for poor gastrointestinal absorption of oral antimicrobials, he was subsequently treated with intravenous tigecycline with an initial, loading dose of 100 mg, followed by 50 mg twice daily for a total of 14 days of therapy. Intravenous doxycycline is not readily available in our institution. His oxygen requirements decreased and fever resolved following 48 hours of treatment with tigecycline. All other antimicrobials were discontinued once the diagnosis of Legionnaires' disease was made. There was no recurrence of infection after three months of follow-up; his repeat chest radiograph showed resolution of his bilateral air space opacities. 

Later in discussion with the North Dakota Department of Health and Centers for Disease Control and Prevention, it was determined that there was an ongoing outbreak of Legionnaires’ disease associated with five cases over a 13-month period; all cases including our patient had stayed at the same hotel. Subsequent environmental testing of the hotel was negative, but this may have been impacted by a recent deep clean of the hotel’s ventilation system.

## Discussion

Legionnaires’ disease and its causative pathogen were first recognized in 1977, following a common-source outbreak of severe pneumonia involving 221 people at an American Legion convention in Philadelphia, Pennsylvania in 1976 [1]. Outbreaks and clusters of cases of Legionnaires’ disease have been associated with contaminated cooling towers, whirlpools, hospital decorative water fountains, hot spring spas, and water births [1-2].

Legionnaires’ disease can be associated with a prodromal illness with symptoms including fever, headache, myalgia, and anorexia; however, the clinical presentation of Legionnaires’ disease is often nonspecific and difficult to distinguish from other causes of community-acquired pneumonia [1-2]. Blood and sputum cultures are relatively insensitive in diagnosing Legionnaires’ disease; in contrast, urine antigen testing has a sensitivity of 60%-95% and specificity greater than 99% [1]. Urine antigen testing for *Legionella* only detects *Legionella* pneumophila serogroup 1 (Lp1) and is most sensitive for the detection of the Pontiac subtype of Lp1, which causes the majority of cases of community-acquired Legionnaires’ disease [1]. *Legionella* urine antigen testing will often be positive on the first day of illness and remain positive for several weeks [1]. Molecular testing of lower respiratory tract specimens can also be used to identify both *Legionella pneumophila* and *Legionella* species by PCR [1].

Preferred therapies for immunocompromised patients with Legionnaires’ disease include levofloxacin and azithromycin [1-3]. Tigecycline is a third generation, intravenous glycylcycline and minocycline derivative that inhibits bacterial protein synthesis by binding to bacterial 30S ribosomal subunits [4]. Prior in vitro and animal model studies have shown that tigecycline achieves high intracellular concentrations [5]. However, demonstrated clinical effectiveness of tigecycline in the treatment of community-acquired pneumonia in humans with Legionnaires’ disease remains limited.

Two prior case reports describe successful use of tigecycline in the treatment of immunocompromised patients with legionellosis; however, fluoroquinolones were used as initial therapy in both of these cases and tigecycline was later added to their antimicrobial regimen [6-7]. A recently published case series describes eight patients with Legionnaires’ disease who were switched to tigecycline, often due to worsening sepsis and/or respiratory status, following initial exposure to macrolide and/or fluoroquinolone therapy (median of three days) [8]. All but one of these eight patients received combination therapy with tigecycline plus either levofloxacin or azithromycin as part of their treatment regimen once tigecycline was added. Furthermore, the one patient in this case series who received 14 days of tigecycline monotherapy had received eight days of azithromycin prior to switching therapy. Thus, it is difficult to ascertain whether clinical improvement in these cases was due to the addition of tigecycline or post-antibiotic effect and delayed response from fluoroquinolone/macrolide therapy.

Integrated results from two randomized controlled trials showed comparable cure rates between tigecycline and levofloxacin in the treatment of hospitalized patients with community-acquired pneumonia, of which a small proportion were diagnosed with Legionnaires’ disease in each treatment arm [9]. While the integrated results of these two randomized controlled trials support the early use of tigecycline as empiric treatment of community-acquired pneumonia, one of these trials permitted switching to oral levofloxacin following at least three days of intravenous therapy if evidence of clinical improvement.

Current evidence, albeit limited, suggests that tigecycline may be added as combination therapy in severe cases of Legionnaires’ disease. This case, however, demonstrates that tigecycline can be effective as a second-line treatment option for Legionnaires’ disease in the setting of allergies to traditional mainstays of therapy. In 2013, the Food and Drug Administration (FDA) approved a new boxed warning about the higher risk of death among patients receiving tigecycline compared with other antibiotics, particularly apparent for hospital-acquired pneumonia and ventilator-associated pneumonia [10]. While both the FDA and Health Canada have approved tigecycline for treatment of community-acquired bacterial pneumonia, complicated skin and soft tissue infections, and complicated intra-abdominal infections, its use should be reserved for situations when alternative treatments are not suitable [10-11]. 

## Conclusions

Legionnaires’ disease is a rare cause of community-acquired pneumonia but can be associated with significant morbidity and mortality, especially amongst immunocompromised individuals. Although the evidence regarding the use of tigecycline in treating Legionnaires' disease is limited, this case report provides evidence supporting the use of tigecycline as a second-line therapeutic option in select cases where fluoroquinolone or macrolide therapy may be contraindicated. 
